# Mode Conversion Behavior of Guided Wave in a Pipe Inspection System Based on a Long Waveguide

**DOI:** 10.3390/s16101737

**Published:** 2016-10-19

**Authors:** Feiran Sun, Zhenguo Sun, Qiang Chen, Riichi Murayama, Hideo Nishino

**Affiliations:** 1 Department of Mechanical Engineering, Tsinghua University, Beijing 100084, China; sfr13@mails.tsinghua.edu.cn (F.S.); chenq@tsinghua.edu.cn (Q.C.); 2 Yangtze Delta Region Institute of Tsinghua University, Jiaxing 314006, China; 3 Department of Intelligent Mechanical Engineering, Fukuoka Institute of Technology, Fukuoka 811-0295, Japan; murayama@fit.ac.jp; 4 Graduate School of Advanced Technology and Science, Tokushima University, Minami-Josanjima, Tokushima 770-8506, Japan; hidero.nishino@tokushima-u.ac.jp

**Keywords:** mode conversion, waveguide, guided wave, EMAT, pipe inspection

## Abstract

To make clear the mode conversion behavior of S0-mode lamb wave and SH0-plate wave converting to the longitudinal mode guided wave and torsional mode guided wave in a pipe, respectively, the experiments were performed based on a previous built pipe inspection system. The pipe was wound with an L-shaped plate or a T-shaped plate as the waveguide, and the S0-wave and SH0-wave were excited separately in the waveguide. To carry out the objective, a meander-line coil electromagnetic acoustic transducer (EMAT) for S0-wave and a periodic permanent magnet (PPM) EMAT for SH0-wave were developed and optimized. Then, several comparison experiments were conducted to compare the efficiency of mode conversion. Experimental results showed that the T(0,1) mode, L(0,1) mode, and L(0,2) mode guided waves can be successfully detected when converted from the S0-wave or SH0-wave with different shaped waveguides. It can also be inferred that the S0-wave has a better ability to convert to the T(0,1) mode, while the SH0-wave is easier to convert to the L(0,1) mode and L(0,2) mode, and the L-shaped waveguide has a better efficiency than T-shaped waveguide.

## 1. Introduction

Online monitoring of pipes at high temperature for wall thickness and defects is a key to many industrial non-destructive evaluation (NDE) demands, such as the power generation and the nuclear industry. It is quite cost effective to avoid plant shutdowns during monitoring of its structures; whereas “high-temperature monitoring” allows us to conduct the plant structure monitoring without shutting down the plant [1].

In recent years, the ultrasonic guided wave testing method has been widely applied for pipe inspections as it carries major advantages such as low attenuation, long distance propagation, and high detection efficiency [2,3,4,5,6]. There are two techniques which are commonly employed for generating guided waves: the piezoelectric transducer and electromagnetic acoustic transducer (EMAT) [2]. However, the piezoelectric ultrasonic testing requires a good sonic contact with the test piece, which restricts its inspection ability for certain applications, especially under high-temperature conditions [7,8]. EMAT has the ability to generate and detect ultrasonic waves without making a contact with the test object which makes it suitable to inspect high-temperature pipes with a large lift-off. Even though the EMAT still requires a cooling system which causes many issues [9,10,11], in addition, the lift-off effect also influences the results [12,13,14,15].

**Figure 1:**
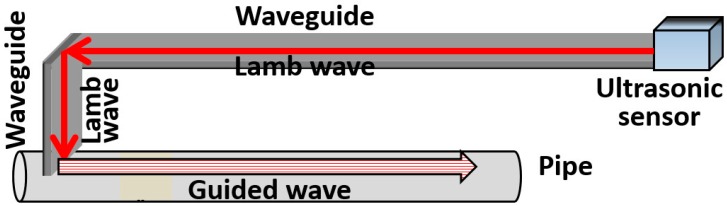
Pipe inspection system using a long waveguide [16].

**Figure 2:**
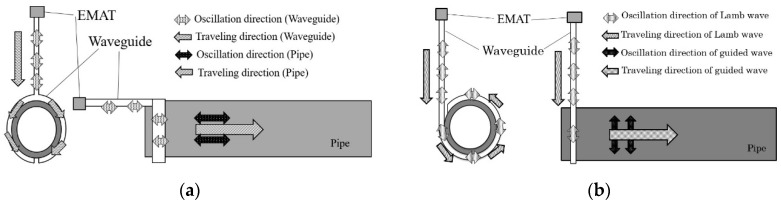
Pipe inspection system with different shaped waveguides based on S0-mode lamb wave (**a**) longitudinal mode with T-shaped waveguide; (**b**) torsional mode with L-shaped waveguide [16].

Therefore, a sensor system for high-temperature pipe inspection, which can transmit and receive ultrasonic guided waves using a long waveguide, has been developed by Murayama [16]. As shown in Figure 1, the tested pipe was wound with a long plate as the waveguide, which could keep the ultrasonic sensor away from the high-temperature pipe. The S0-mode lamb wave was excited in the plate-waveguide as the source, and it has been proved that the S0-wave can convert to the longitudinal mode guided wave in the pipe with the T-shaped waveguide and to the torsional mode guided wave with the L-shaped waveguide [16], as shown in Figure 2. However, the longitudinal and torsional mode guided waves have different detection abilities for different types of defects due to their different oscillation patterns, thus both of them are always employed for a comprehensive pipe evaluation [6,16,17,18]. Therefore, in this system, if the transformation of testing wave from the longitudinal mode to the torsional mode is required, the waveguide will be converted from the T-shaped to the L-shaped fixed on the high-temperature pipe, which cannot be easily executed in practice.

As a consequence, based on the already established pipe inspection system, a method to change the mode of the testing wave in the pipe by replacing the S0-wave EMAT with SH0-wave EMAT was proposed in this research, which was much easier than removing the fixed waveguide. An SH0-wave periodic permanent magnet (PPM) EMAT and an S0-wave meander-line coil EMAT were developed, then the experiments were conducted involving the mode conversion from SH0 mode and S0 mode guided waves to the longitudinal and torsional mode guided waves, respectively. In addition, the mode conversion behavior for each mode of guided wave was studied by calculating and comparing the mode conversion efficiency, from SH0 and S0 to longitudinal and torsional, respectively.

## 2. Method

In order to test a pipe with longitudinal and torsional mode guided waves, based on the previous built pipe inspection system, a method to change the mode of the testing wave in the pipe by changing the EMAT on the waveguide without removing the fixed waveguide was proposed. That is, the T-shaped waveguide and L-shaped waveguide would not be switched with each other, and the S0-mode EMAT needed to be changed to the other mode guided wave sensor to complete the switch between the longitudinal mode and torsional mode, which was much easier to implement in an industrial NDE. Thus, the key was to find another mode guided wave in the plate-waveguide which could separately convert to the longitudinal and torsional mode guided waves using the opposite waveguide of the S0-mode.

### 2.1. Guided Wave into a Pipe

**Figure 3:**
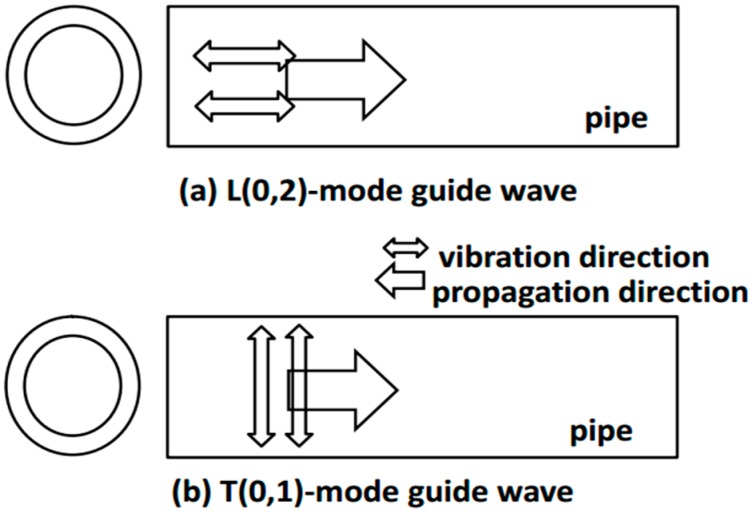
Oscillation patterns of the (**a**) L(0,2) and (**b**) T(0,1) mode guided waves in pipes [16].

The axially symmetric L(0,2) and T(0,1) modes are the most widely-used modes for pipelines [19]. The oscillation patterns of the L(0,2) and T(0,1) mode guided waves are shown in Figure 3, in which the oscillation direction of L(0,2) is parallel to its propagation direction, but for T(0,1) mode, the oscillation direction is perpendicular to the propagation [16].

**Figure 4:**
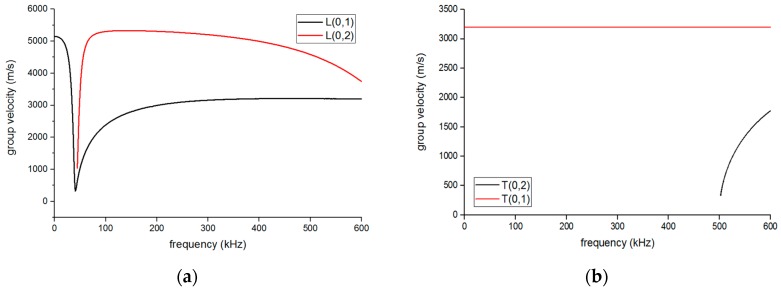
The dispersion curves of group velocity in the pipe: (**a**) longitudinal mode and (**b**) torsional mode.

**Figure 5:**
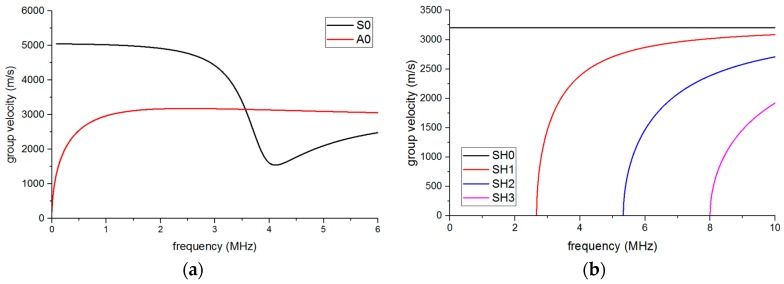
The dispersion curves of group velocity in the waveguide: (**a**) lamb wave and (**b**) shear horizontaL(SH) wave.

Then, the dispersion curves of the pipe and the plate-waveguide were built using a numerical method with MATLAB based on an analysis of the dispersion equations [20,21], as shown in Figure 4 and Figure 5. The relevant parameters of the pipe and waveguide can be obtained from Table 1.

If the T(0,1) mode is one of the objective guided waves, the exciting frequency should be below the cut-off frequency of 500 kHz to eliminate the other interference torsional modes in Figure 4b. Based on Figure 4a, the L(0,1) mode guided wave may exist during the test even if we try to avoid it through the design of the EMAT, for it has the same frequency range as the L(0,2) mode. However, at the frequency of around 100 kHz, the group velocity of L(0,2) is much higher than that of L(0,1) so that we can remove the L(0,1) part by reception signal selection [22]. Therefore, the exciting frequency was chosen as 100 kHz and the wavelengths of S0 and SH0 could be computed as 50 mm and 32 mm, respectively, from Figure 5, which can help in the design of the EMAT.

### 2.2. Method and Assumption

Based on Figure 2a,b, it can be noted that the oscillation direction remains unchanged when the S0-mode guided wave travels from the waveguide to the pipe no matter which shaped waveguide is used. The reason for generation of different modes of guided waves in the pipe is the different connection patterns of the different shaped waveguides, that is the T-shaped and L-shaped waveguides. The T-shaped waveguide is parallel to the axis of the pipe so that the consistent oscillation direction is parallel to the axis and the propagation direction is always along the axis, thus forming the L(0,2) mode. This is the same for the L-shaped waveguide and T(0,1) mode.

**Figure 6:**
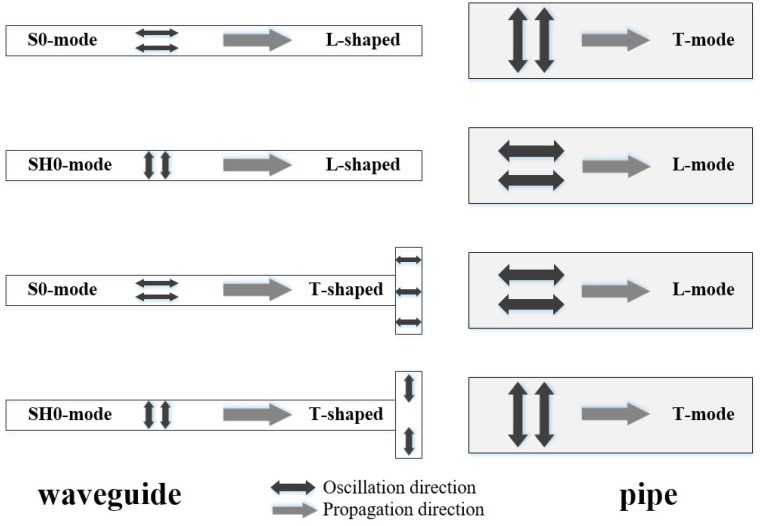
The mode conversion experiments to prove the assumption.

Consequently, based on this phenomenon, it can be assumed that a guided wave whose oscillation direction is perpendicular to its propagation in a plate can convert to the T(0,1) mode guided wave using the T-shaped waveguide or L(0,2) mode using the L-shaped waveguide. That is, the shear horizontaL(SH) wave is a polarized guided wave in-plane with respect to the reference interface [23]. There are multiple symmetric and anti-symmetric SH wave modes. This study used only the lowest order SH mode, SH0, which is a symmetric dispersionless mode [24] just like the S0 mode. Based on this assumption, experiments were performed involving the mode conversions from S0-mode and SH0-mode to T-mode and L-mode, respectively, as shown in Figure 6.

## 3. Experimental System

**Figure 7:**
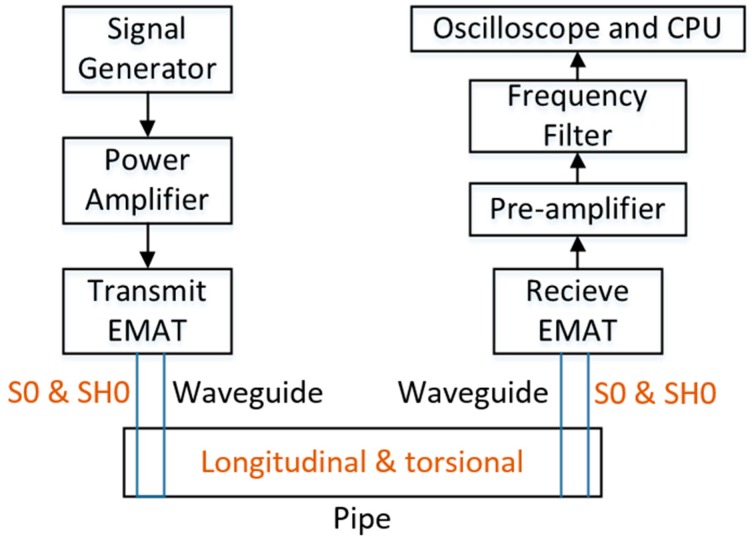
Block diagram of experimental system.

**Figure 8:**
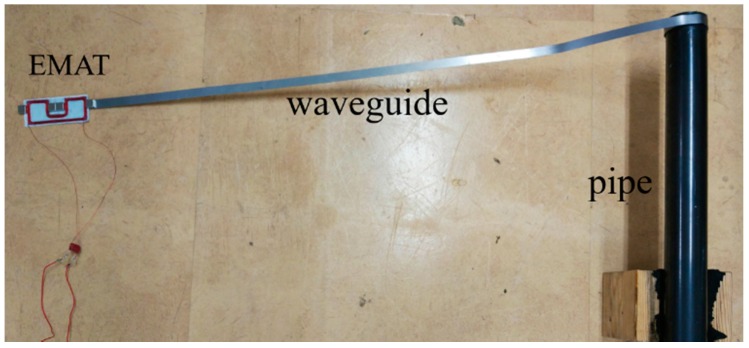
The photo of the pipe inspection system.

As shown in Figure 7, the experimental system consists of a function generator and a power amplifier, which generates the 100 kHz frequency and six volt amplitude electric power with four cycles of burst shape, and is amplified 20 times, then input to an electromagnetic induced coil, a pre-amplifier and a frequency filter, which amplifies the received signal by 50 dB, and selects the signal between 50 kHz and 200 kHz, and an oscilloscope and central processing unit (CPU), which collects and evaluates the received signal. The two waveguides of 1 m long, 10 mm wide and 0.6 mm thick plate were separately connected around the pipe, and the transmit EMAT and receive EMAT were placed on the terminal of the waveguides. Figure 8 shows the part of the experimental system which consists of the EMAT and the waveguide winding around the test pipe.

## 4. Development and Optimization of EMATs

### 4.1. Meander-Line Coil EMAT for S0 Mode Wave

**Figure 9:**
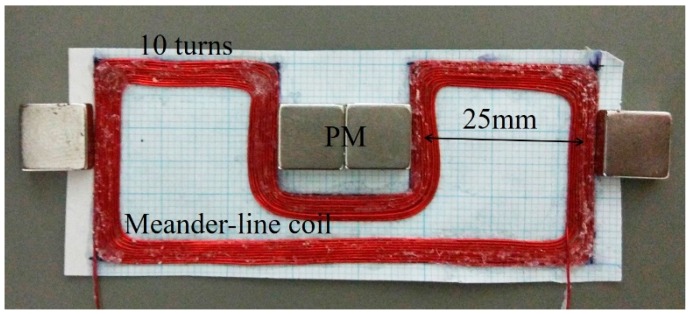
The structure of meander-line coil EMAT for S0-mode guided wave.

The meander-line coil EMAT has been widely used to excite a lamb wave in a plate-waveguide [25]. In this study, permanent magnets (PM) were used to provide a static magnetic field. The PM was 10 mm in length, 10 mm in width and 4 mm in thickness, and the magnetic field density at the surface was 326.4 mT. As shown in Figure 9, the meander-line coil was made of copper wire with a diameter of 0.315 mm, and the pitch between the meander lines was equal to half the wavelength of 25 mm according to the drive principle.

**Figure 10:**
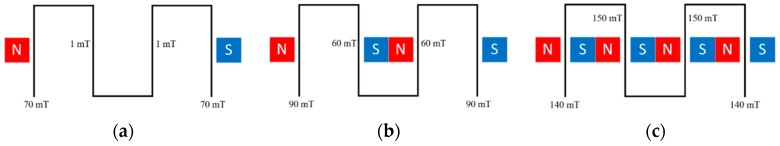
Three configurations of permanent magnets (PMs) of EMAT: (**a**) two magnets; (**b**) four magnets; and (**c**) eight magnets.

In order to excite a pure S0-mode lamb wave in the waveguide, the meander-line coil EMAT was optimized. First of all, to produce the best static magnetic field, the configuration of the permanent magnets above the coil was tested in the free waveguide whose dimensions were presented in Table 1. The transmit EMAT and receive EMAT were separately placed on each side of the waveguide, and there were three reasonable configurations of the PMs to be tested as shown in Figure 10. The densities of the static magnetic field were measured in these three configurations using a gauss meter, also shown in Figure 10.

**Figure 11:**
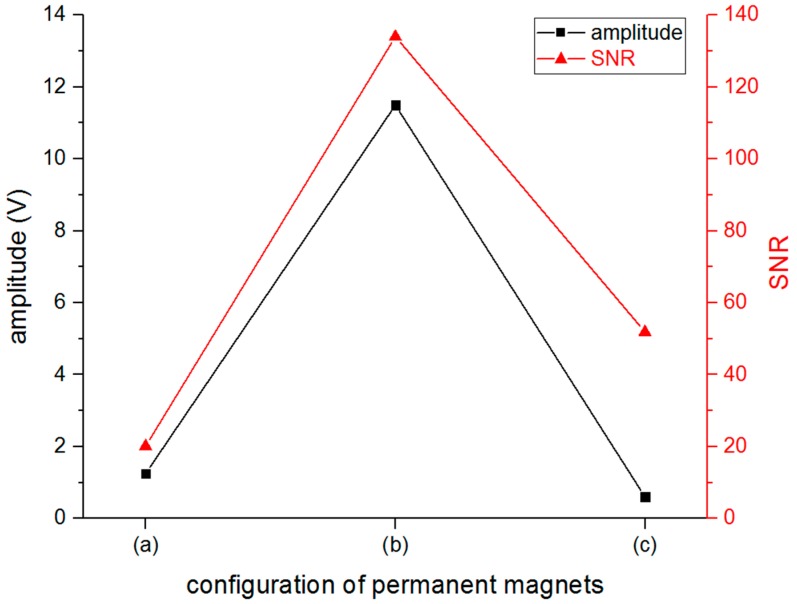
The amplitude and signal to noise rate (SNR) value of S0 wave with different configurations of PMs shown in Figure 10.

The amplitude and signal to noise rate (SNR) value of the S0-wave were computed from the received signal, then, these three configurations were compared to determine the best one, just as in Figure 11. It indicated that the amplitude and SNR could reach the highest values when the 4-PM configuration was selected from these three candidates.

**Figure 12:**
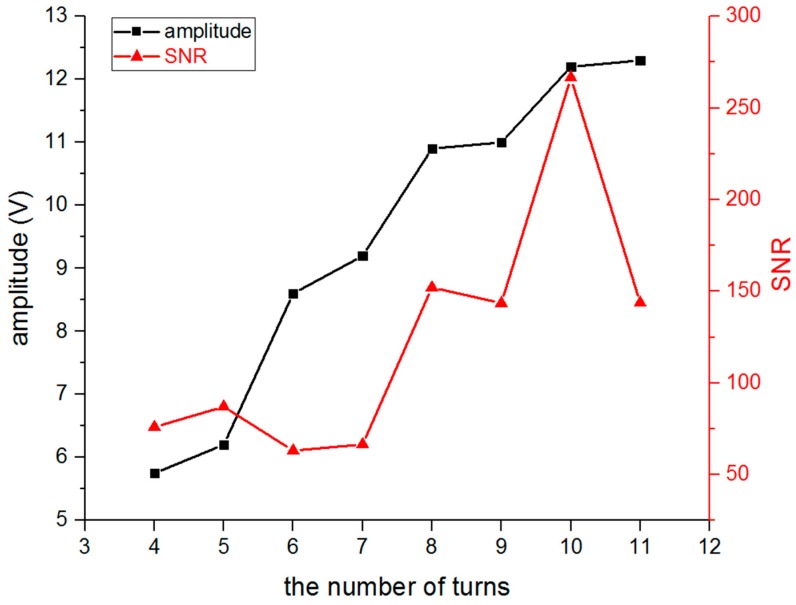
The amplitude and SNR of S0 wave with a different number of turns of meander-line coil.

Next, the number of turns of the meander-line coil was optimized. The test was conducted while changing the number of turns from four to eleven, and the capacitance, inductance, resistance, and impedance of each coil were measured using an LCR (inductance, capacitance, resistance) meter as shown in Table 2. Figure 12 shows the relationship of how the amplitude and SNR of the S0 wave varied while the number of turns increased. It also shows that the amplitude increased with the increasing number of turns, and the SNR obtained the maximum value when the number of turns was equal to ten.

As a result, an EMAT with a 10-turn meander-line coil and 4-PM configuration has been developed as shown in Figure 9, which has the best drive conditions according to Figure 11 and Figure 12.

### 4.2. PPM EMAT for SH0-Mode Wave

**Figure 13:**
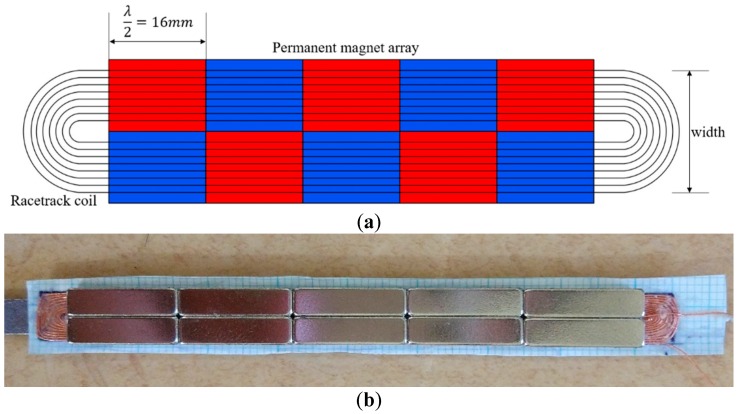
(**a**) The layout of PPM EMAT; (**b**) The outlook of PPM EMAT for SH0 wave.

To generate an SH0-mode guided wave, the periodic permanent magnet (PPM) EMAT [26,27,28] was employed in this study. The PPM EMAT consisted of a racetrack-shaped coil with a magnet array on top (two columns and several rows of alternating polarity magnets) as shown in Figure 13. The racetrack coil had a 5 mm width with eight turns of 0.2 mm diameter wire. The permanent magnet was as long as half the wavelength of the SH0 wave, and the magnetic field density at the surface was 281.1 mT.

**Figure 14:**
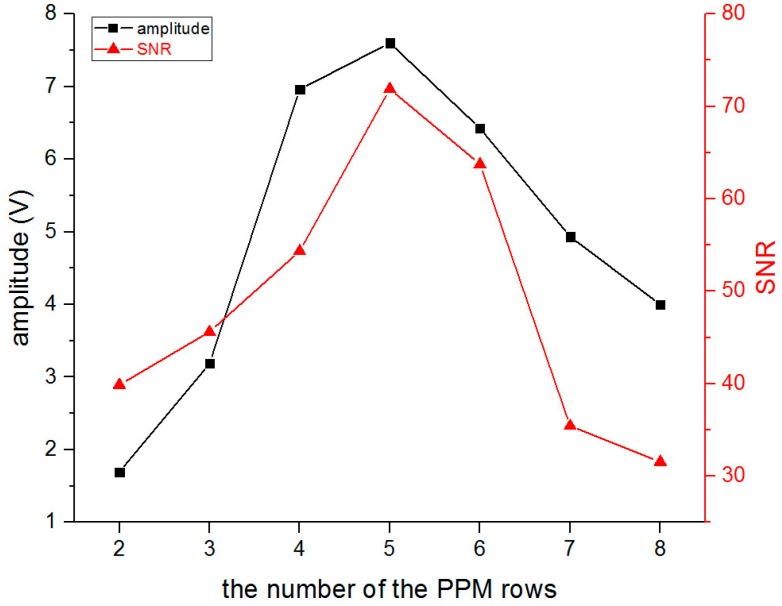
The amplitude and SNR of SH0 wave with different number of PM rows.

Similar to the optimization tests for the meander-line coil EMAT, the number of PPM rows was optimized to obtain a good SNR signal. The test was conducted while changing the number of PPM rows from two to eight, and accordingly, the length of the racetrack coil had to be changed to fit the length of the PPM array, and the capacitance, inductance, resistance, and impedance of each coil were measured using an LCR meter as shown in Table 3. Figure 14 shows the result of the amplitude and SNR of the SH0 wave while the number of PPM rows was set from two to eight. And it is easy to find that when the number of PPM rows is equal to five, the PPM EMAT can generate the best SNR SH0 wave.

## 5. Experiment to Detect a Guided Wave into the Pipe Using the S0 and SH0 Waves

**Figure 15:**
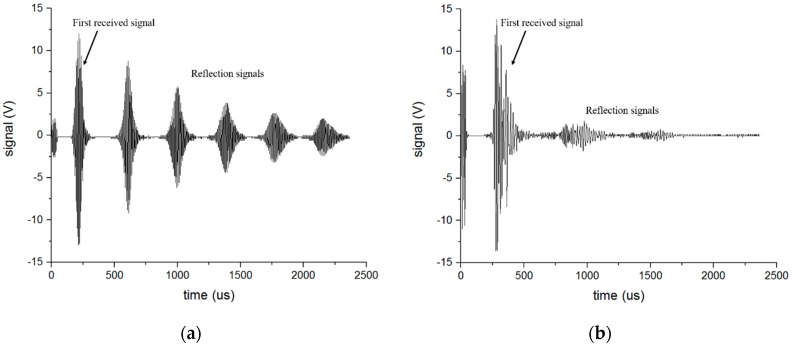
The received signal in the free waveguide: (**a**) S0-wave; (**b**) SH0-wave.

In order to select the appropriate parameters for the device, such as the function generator and amplifier, as well as to compute the mode conversion efficiency later, we conducted an experiment for the S0 wave and SH0 wave in the free waveguide before traveling to the pipe. The transmit EMAT and receive EMAT were separately placed on each side of the waveguide so that it could receive ideal signals of the S0 wave and SH0 wave through parameter selection as shown in Figure 15. From Figure 15a,b, the velocities of the S0 wave and SH0 wave were separately computed to be about 5000 m/s and 3200 m/s, respectively, that agreed well with the group velocity calculated from the dispersion curve. The amplitude of the first received signal of the S0 and SH0 was measured as 12.10 V and 13.75 V, respectively.

**Figure 16:**
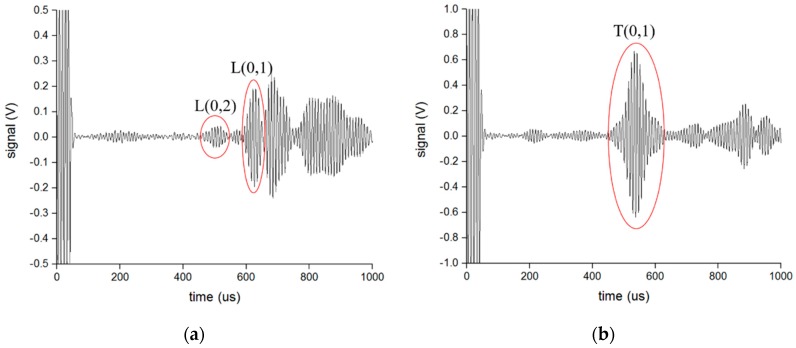
The received signal converted from S0 wave when the distance between the waveguides is 0.5 m: (**a**) Longitudinal wave using T-shaped waveguide; (**b**) Torsional wave using L-shaped waveguide.

**Figure 17:**
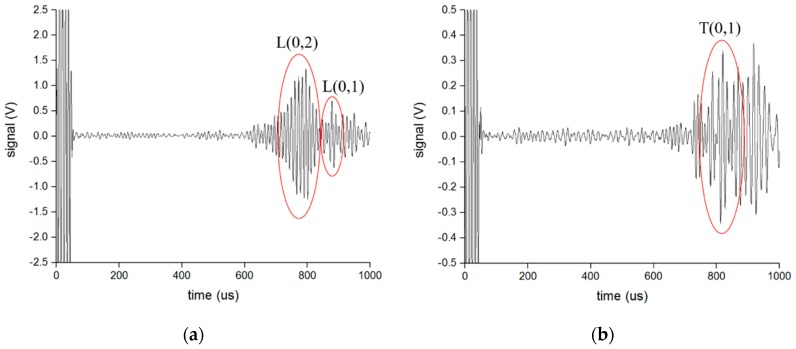
The received signal converted from SH0 wave when the distance between the waveguides is 0.5 m: (**a**) Longitudinal wave using L-shaped waveguide; (**b**) Torsional wave using T-shaped waveguide.

The experiments were then processed in the mode conversion from S0 and SH0 to the longitudinal mode and torsional mode guided waves, respectively, using the opposite waveguide to confirm the previous assumption. The distance between the two waveguides was set at 0.5 m, 1 m, and 1.5 m to verify the received results. Figure 16 and Figure 17 present an example of the received signals converted from the S0 wave and SH0 wave, respectively, when the distance between two waveguides is 0.5 m and the length of the waveguide is 1 m. The velocities of the L(0,2) mode, L(0,1) mode, and T(0,1) mode are about 5200 m/s, 2300 m/s, and 3200 m/s, respectively, from the developed dispersion curves. We could then compute the transmit time for each mode of guided wave and speculate which waveform was the objective mode as labeled in the Figure 16 and Figure 17. This was the same with the signals when the distance between the waveguides was 1 m and 1.5 m. From the received signals, it can be proven that the SH0-mode guided wave can successfully convert to the longitudinal mode and torsional mode guided waves with the opposite waveguide of the S0-wave, and the method of shifting the EMATs to change the mode of the guided wave in the pipe can be implemented.

**Figure 18:**
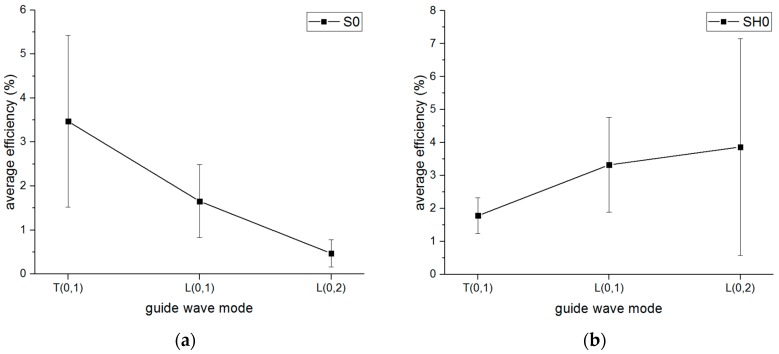
The efficiency of mode conversion to different mode guided waves in the pipe (**a**) converted from S0 and (**b**) converted from SH0.

Furthermore, the amplitude of each mode of guided wave could be measured from the received signal, then the mode conversion efficiency could be calculated, which is defined as the ratio of the amplitude of the guided wave in the pipe to the amplitude of the first received signal in the free waveguide. The average amplitude of the guided wave for the three distances between the waveguides was used. The results of the mode conversion to the different mode guided waves in the pipe from the S0-wave and SH0-wave are shown in Figure 18. It can be observed that the mode conversion from the S0-wave to the T(0,1) mode guided wave has the best efficiency, and the efficiency of the S0-wave converting to the L(0,2) mode has the lowest efficiency. However, it is quite the opposite for the efficiency result converted from the SH0 wave, in other words, the SH0 wave is most likely to convert to the L(0,2) mode rather than the T(0,1) mode.

**Figure 19:**
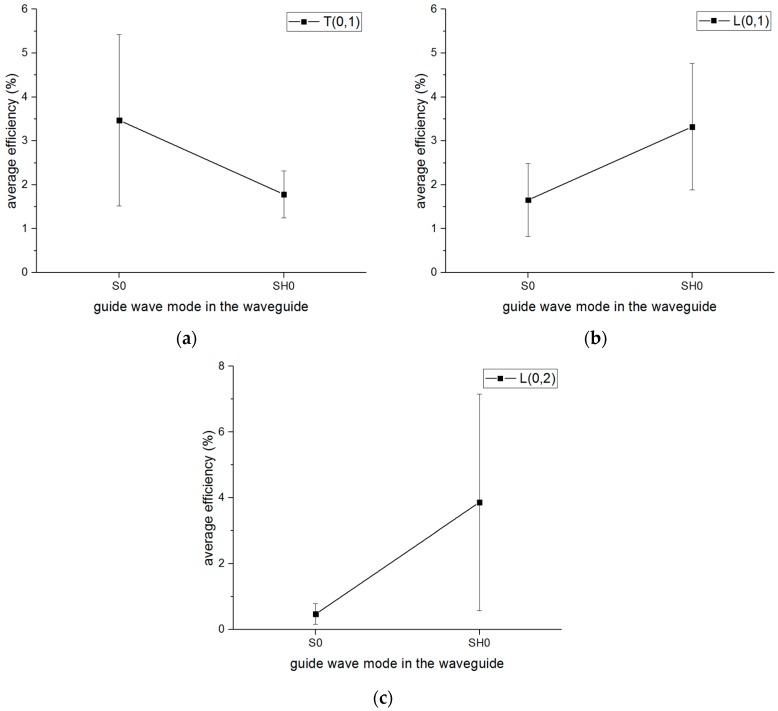
The efficiency comparison between S0 wave and SH0 wave when they convert to the same mode guided wave in the pipe: (**a**) T(0,1) mode; (**b**) L(0,1) mode; (**c**) L(0,2) mode.

It is also necessary to compare the mode conversion behaviors between the S0-wave and SH0-wave when they convert to the same mode guided wave in the pipe shown in Figure 19. Therefore, it can be concluded that the S0-wave has a better efficiency to convert to the T(0,1) mode guided wave than the SH0-wave, while the SH0-wave is more likely to convert to the L(0,1) and L(0,2) mode guided waves than the S0-wave, which is a very important characteristic of the mode conversion behavior.

**Figure 20:**
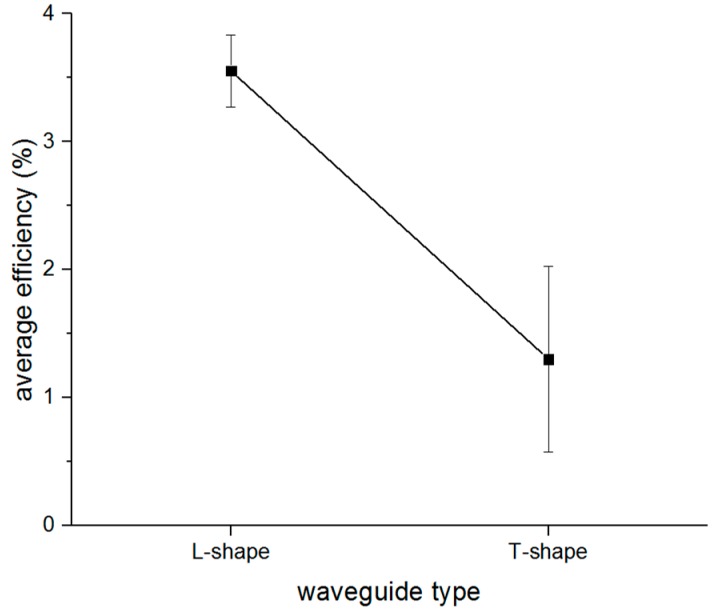
The efficiency comparison between two waveguides.

In addition, the average efficiency of the different mode conversions could be calculated when the same waveguide was used. For example, the average efficiency of mode conversions including S0 to T(0,1), SH0 to L(0,1), and SH0 to L(0,2) can be regarded as the efficiency for the L-shaped waveguide, and also the average efficiency for the T-shaped waveguide. The comparison between the efficiency of the two waveguides is shown in Figure 20, which indicates that the L-shaped waveguide had a better efficiency than the T-shaped waveguide no matter which mode guided wave was excited in the waveguide.

## 6. Conclusions

In this study, based on the previous pipe inspection system, an SH0-wave PPM EMAT and an S0-wave meander-line coil EMAT were developed and optimized based on the dispersion curves of the guided waves. The experiments were successfully performed on the mode conversion from the SH0-mode to the longitudinal modes with the L-shaped waveguide and to the torsional mode with the T-shaped waveguide. The results showed that the mode switch between the longitudinal and torsional can be accomplished by the shift between the S0-EMAT and SH0-EMAT without shifting the fixed waveguide, which will provide significant convenience in the process of pipe evaluations. Moreover, the mode conversion behaviors were studied by calculating and comparing the amplitude efficiency. It can be inferred that the S0 wave has a better ability to convert to the T(0,1) mode, while the SH0 wave has a better ability to convert to the L(0,1) mode and L(0,2) mode, and the L-shaped waveguide has better efficiency than the T-shaped waveguide.

## Figures and Tables

**Table 1:** The parameters of the pipe and waveguide.

Parameter	Pipe	Waveguide (Plate)
Inner radius (width of plate)/mm	18.195	10
Thickness/mm	3.22	0.6
Velocity of shear wave/(m/s)	3200	3200
Velocity of longitudinal wave/(m/s)	5940	5200

**Table 2:** The capacitance, inductance, resistance, and impedance of the meander-line coil.

Number of Turns	Capacitance/nF	Inductance/μH	Resistance/mΩ	Impedance/Ω
4	822.77	2.7921	289.45	1.7815
5	652.10	3.5233	318.75	2.3468
6	462.17	4.9716	386.84	3.3027
7	371.51	6.1839	439.51	4.1030
8	280.44	8.1950	506.31	5.4305
9	238.35	9.6395	533.51	6.3814
10	200.12	11.481	626.32	7.6005
11	176.87	12.989	656.48	8.5942

**Table 3:** The capacitance, inductance, resistance, and impedance of the racetrack coil.

Number of the PPM Rows	Capacitance/nF	Inductance/μH	Resistance/mΩ	Impedance/Ω
2	1305.3	1.9406	494.69	1.3159
3	859.75	2.9478	730.01	1.9891
4	734.08	3.4506	831.21	2.3216
5	584.11	4.3366	955.75	2.8874
6	503.73	5.0169	1156.0	3.3643
7	429.12	5.9027	1271.4	3.9403
8	370.45	6.8380	1445.7	4.5329

## References

[1] Burrows S.E., Fan Y., Dixon S. (2014). High temperature thickness measurements of stainless steel and low carbon steel using electromagnetic acoustic transducers. NDT E Int..

[2] Liu Z., Hu Y., Fan J. (2016). Longitudinal mode magnetostrictive patch transducer array employing a multi-splitting meander coil for pipe inspection. NDT E Int..

[3] Duan W., Kirby R. (2015). A numerical model for the scattering of elastic waves from a non-axisymmetric defect in a pipe. Finite Elem. Anal. Des..

[4] Willey C.L., Simonetti F., Nagy P.B. (2014). Guided wave tomography of pipes with high-order helical modes. NDT E Int..

[5] Nagy P.B., Simonetti F., Instanes G. (2014). Corrosion and erosion monitoring in plates and pipes using constant group velocity Lamb wave inspection. Ultrasonics.

[6] Leinov E., Lowe M.J.S., Cawley P. (2016). Ultrasonic isolation of buried pipes. J. Sound Vib..

[7] Cegla F.B., Jarvis A.J.C., Davies J.O. (2011). High-temperature ultrasonic crack monitoring using SH waves. NDT E Int..

[8] Cegla F.B., Cawley P., Allin J. (2011). High-temperature (>500 °C) wall thickness monitoring using dry-coupled ultrasonic waveguide transducers. IEEE Trans. Ultrason. Ferroelectr. Freq. Control.

[9] Murayama R., Kobayashi M., Matsumoto K. (2014). Ultrasonic Inspection System Using a Long Waveguide with an Acoustic Horn for High-Temperature Structure. J. Sens. Technol..

[10] Hernandez-Valle F., Dixon S. (2009). Preliminary tests to design an EMAT with pulsed electromagnet for high temperature. Am. Inst. Phys..

[11] Hernandez-Valle F., Dixon S. (2010). Initial tests for designing a high temperature EMAT with pulsed electromagnet. NDT E Int..

[12] Tian G.Y., Sophian A. (2005). Reduction of lift-off effects for pulsed eddy current NDT. NDT E Int..

[13] Huang S., Zhao W., Zhang Y. (2009). Study on the lift-off effect of EMAT. Sens. Actuators A Phys..

[14] Yu Y., Yan Y., Wang F. (2014). An approach to reduce lift-off noise in pulsed eddy current nondestructive technology. NDT E Int..

[15] Morrison J.P., Dixon S., Potter M.D.G. (2006). Lift-off compensation for improved accuracy in ultrasonic lamb wave velocity measurements using electromagnetic acoustic transducers (EMATs). Ultrasonics.

[16] Murayama R., Matsumoto K., Ushitani K. (2015). Pipe Inspection System by Guide Wave Using a Long Distance Waveguide. Mod. Mech. Eng..

[17] Lee H., Park H.W., Sohn H. (2014). Pipe Defect Visualization and Quantification Using Longitudinal Ultrasonic Modes. Int. J. Struct. Stab. Dyn..

[18] Murayama R., Weng J., Kobayashi M. (2014). Pipe inspection system of a pipe by three-modes guide wave using polarized-transverse wave EMATs. Proc. SPIE.

[19] Xu J., Wu X., Kong D. (2015). A Guided Wave Sensor Based on the Inverse Magnetostrictive Effect for Distinguishing Symmetric from Asymmetric Features in Pipes. Sensors.

[20] Zheng X.M., Zhao Y.Z., Shi Y.W. (2003). Calculation for lamb wave dispersion curves. Nondestr. Test..

[21] Xu X.N., Yu Z.J., Zhu L.Q. (2012). A graphical method to solve a dispersion equation of Lamb wave. J. Electron. Meas. Instrum..

[22] Lowe M.J.S., Alleyne D.N., Cawley P. (1998). Defect detection in pipes using guided waves. Ultrasonics.

[23] Murayama R., Makiyama S., Kodama M. (2004). Development of an ultrasonic inspection robot using an electromagnetic acoustic transducer for a Lamb wave and an SH-plate wave. Ultrasonics.

[24] Petcher P.A., Burrows S.E., Dixon S. (2014). Shear horizontaL(SH) ultrasound wave propagation around smooth corners. Ultrasonics.

[25] Murayama R., Mizutani K. (2002). Conventional electromagnetic acoustic transducer development for optimum Lamb wave modes. Ultrasonics.

[26] Andruschak N., Saletes I., Filleter T. (2015). An NDT guided wave technique for the identification of corrosion defects at support locations. NDT E Int..

[27] Petcher P.A., Dixon S. (2015). Weld defect detection using PPM EMAT generated shear horizontal ultrasound. NDT E Int..

[28] Hill S., Dixon S. (2014). Frequency dependent directivity of periodic permanent magnet electromagnetic acoustic transducers. NDT E Int..

